# Case of Imperforate Hymen

**Published:** 1800

**Authors:** Henry Fryer

**Affiliations:** Surgeon at Stamford.


### X. *Case of imperforate Hymen. By the same.*

DECEMBER 28th, 1796, I was sent for in a great hurry to A. B. of this place, who, as I was informed, had not voided any urine for several hours. I was, at the same time, told that she had been very much out of health for some months past, having had great pain in making water and going to stool; and that there was also a large tumor between the labia pudendi, which had been supposed to be a prolapsus uteri.

Upon going into the room, I found her making heavy complaints, and, therefore, immediately proceeded to draw off the water, which I had no difficulty in doing. I afterwards examined the tumor, and perceived that I could not pass my finger at all into the vagina. It then first struck me that the hymen was imperforated, and, upon enquiry, I made out that her pains had returned at different times, after intervals of three weeks or a month; that she was now sixteen years old, and that her breasts had been full for some time; she had also every other appearance of puberty. The cafe appearing fo extraordinary, I fent for my friend, Doctor Cooper, of this place, who, upon examination, agreed with me in opinion, that all her complaints had arifen from the confinement of the menftrual fluid. Indeed, that peculiar feel from fluctuation of blood, which can be known by thofe alone who have frequently felt it, put it beyond all doubt; we, therefore, determined immediately upon the operation; and, upon introducing a large abfcefs lancet, with a violent gufh, fet at liberty full thirty ounces of fluid by weight, befides what was loft in the bed. I then paffed in a probe-pointed knife upon the fore finger of my left hand, and divided the fkin freely upwards and downwards; and after having thoroughly cleaned her, introduced a piece of linen cloth, rolled up hard, and fmeared with lard, into the vagina, until we had time to prepare a better peffary; this was kept in by the help of a bandage. The next day, I contrived a peffary, made of the barrel of an ivory fyringe, covered with flax, and dipped in melted wax, which anfwered perfectly well, being taken out occafionally, wafhed and replaced. From this time every bad fymptom left her; fhe

ſhe ſoon was quite recovered; and at the end of about ſix weeks the menſtrual diſcharge appeared, which has continued to return at the proper periods ever ſince.

